# Can the Effect of Problem Solvers’ Characteristics on Adolescents’ Cooperative Problem Solving Ability Be Improved by Group Sizes?

**DOI:** 10.3390/ijerph192416575

**Published:** 2022-12-09

**Authors:** Chuanhua Gu, Xiaoqing Ma, Qianqian Li, Chun Li

**Affiliations:** 1 School of Psychology, Central China Normal University, Wuhan 430079, China; 2 Key Laboratory of Adolescent Cyberpsychology and Behavior, Ministry of Education, Wuhan 430079, China; 3 Department of Preschool Education, Qingdao University, Qingdao 266000, China

**Keywords:** cooperative problem solving, group size, problem-solving orientation, problem-solving style, social problem solving

## Abstract

Cooperative problem solving (CPS) is an essential ability in people’s daily life. When individuals with different problem-solvers’ characteristics (orientation and style) are assigned to different group sizes to solve social tasks, what are the differences in the performance of CPS ability? Based on this, through online experimental tasks, the present study examined the effect of problem-solving orientation and style on CPS ability in online social tasks. Meanwhile, it explored the role of group sizes as an environmental variable. The results showed that the more positive the problem-solving orientation, the better the performance of individual CPS ability. In addition, the more rational the problem-solving styles and the larger the group sizes, the higher the scores of participants’ CPS ability. This study provides a new theoretical perspective for the complex relationship between the characteristics of problem solvers and CPS ability, and also provides empirical support for the cultivation of the CPS ability of adolescents.

## 1. Introduction

Cooperation with others has been regarded as the core form of human activities [1], and cooperation is also one of the factors that promote social progress. The social division of labor makes people pay more and more attention to the cultivation of cooperative problem-solving (CPS) ability. The OECD (2017) [2] pointed out that CPS is an essential ability for people in education and work. It refers to the ability of an individual to effectively participate in the problem-solving process of two or more agents. In this process, individuals share the understanding and efforts needed to reach a solution to the problem, and at the same time, they combine their knowledge, skills, and efforts to achieve the solution reached.

In recent years, CPS ability has been widely studied in the field of education. It was found that students’ individual factors (including subject knowledge, personality, emotion, experience, motivation, and cognitive ability) will affect their cooperation and problem-solving process [2,3,4]. However, these individual factors are mainly concentrated in the cognitive dimension, while the individual characteristics in the social dimension (orientation and style) have not been investigated yet. Previous studies have shown that problem situation, task characteristics, and groups composition can affect the type of cooperation and the process of problem solving [2,5,6]. At present, most CPS ability research uses “scientific tasks” as experimental tasks, while there is little research that uses “social tasks” in social fields. “Social tasks” is regarded as the basic task of solving social problems, which covers a wide area and has strong applicability, so the social tasks research should be increased.

General cognitive tasks (i.e., balance problems, memory tasks, conservation tasks, and mathematical problems) are usually used in problem-solving research. The relevant research focuses more on the individual, but research on the individual’s CPS ability in the groups is rarely involved. Group social creativity refers to the ability of group members to cooperate with each other in a specific way and jointly propose or solve social problems in personal life, interpersonal relationships, and social environments. It often shows unique, novel, appropriate, and effective problem-solving strategies. Compared with the independent thinking of individuals, the nature of CPS creativity can be shown in the study of groups accomplishing tasks together.

This study uses the method of an online cooperative problem-solving experiment to investigate the effect of an individual’s problem-solving orientation/style on their CPS ability, and effectively intervene in their CPS ability to find the best conditions to promote their CPS performance, to provide theoretical support for the cultivation of this ability.

## 2. Theoretical Background

### 2.1. Collaborative Problem Solving

People pay more and more attention to the cultivation and promotion of CPS ability [7]. Cooperation is considered to be a kind of coordinated and synchronous activity to continuously establish and maintain a common idea about a certain problem [8]. Problem solving refers to the process in which an individual tries to find a way to achieve the goal based on the present situation in a problem situation where the solution is unclear [9]. With the improvement of the refined level of social division of labor, cooperation between two or more people has become the basic mode to solve problems in various fields, and cooperation and problem solving have gradually been integrated into CPS [10]. 

Compared with individual problem solving, the advantages of CPS are obvious: (1) CPS has a more effective division of labor, which makes the problem solved faster and better; (2) solutions to problems can include knowledge, opinions, and experience from various sources; (3) the ideas of other group members can improve the quality of individual solutions [11]. CPS includes two key dimensions: cognitive dimension and social dimension [9,12]. In the cognitive dimension, group members need to work together, exchange information, form a common understanding of problems, and discuss the most appropriate strategies to solve problems. Then, they should supervise and modify the strategies to solve problems until the group’s goals are achieved. In the social dimension, the main process is group communication, which can promote or hinder the progress of cooperation in the cognitive dimension. 

Combined with previous studies, it is found that most of the empirical studies on the effect factors of adolescents’ CPS ability focus on students’ individual factors: gender and cooperative attitude; family factors: family socioeconomic status; school factors: teacher–student relationship and the teacher’s method of teaching [13,14,15,16,17]. The effect of individual differences on students’ CPS ability is also very different.

### 2.2. Social Problem Solving

Social problem solving is defined as a self-oriented cognitive-behavioral process in which individuals try to identify special problems encountered in daily life and find effective solutions [18]. The structure of social problem-solving skills is multidimensional and multifaceted. D’Zurilla and Goldfried put forward the theoretical model of social problem solving, which was developed and modified by D’Zurilla and Nezu [19]. The theoretical model of social problem solving divides social problem solving into two relatively independent parts: (1) problem-solving orientation; (2) problem-solving styles. Problem-solving orientation includes two dimensions: positive problem-solving orientation and negative problem-solving orientation, while problem-solving styles consist of three dimensions: rational problem-solving styles, impulse/neglect problem-solving styles, and escape problem-solving styles.

Generally, the research on solving social problems can be divided into two categories: the first is theoretical model research [20,21]; the second is applied research [22,23]. The first type of research is represented by the research of D’Zurilla who put forward a five-factor model for solving social problems. In the second type of research, the researchers explored the effectiveness of social problem-solving ability and studied its relationship with specific behaviors and health. On this basis, the related factors of social problem-solving ability were investigated, followed by a series of intervention studies [24,25]. 

### 2.3. Group Sizes

Cooperation is everywhere, and most students’ learning activities in school need to be carried out through interaction with peers. When students solve a problem together, they need to adjust the differences in problem understanding among the members of the group by establishing a common understanding, and they should negotiate the behaviors of the members of the group. Then, they form a solution to the problem and reach a consensus to finally achieve the group goals [26]. Cooperation is the core of this process. 

Previous studies have found that researchers mostly measure the scale of cooperative groups in the form of two-person groups or three-person groups, for example, the measurement of cooperative problem solving in PISA2015 [2]. Moreover, these studies pay more attention to the “problem solving” of cooperative problem-solving ability, but as one of the core dimensions of cooperative problem solving, “the size of the cooperative group” seems to have not been systematically explored by researchers.

## 3. Current Study

According to the analysis of the existing research, it was found that the individual will be different from person to person when solving social problems, which is mainly manifested in the differences in the characteristics of two problem solvers: problem orientation and problem style. Based on previous studies, this study explores the influence of different orientations/styles on teenagers’ CPS ability by two behavioral experiments, and finds ways to promote individuals’ CPS ability.

## 4. Experiment 1

### 4.1. Hypotheses

Previous studies have indicated that the performance of individual CPS ability is mainly focused on individual factors, family economic status, and the relationship between teachers and students [13,14,27]. Experiment 1 was intended to investigate the effect of problem-solving orientation (positive and negative) and the number of group sizes (small and large) on CPS ability. We proposed the following hypotheses:

**H1.**  *Problem-solving orientation affects participants’ CPS ability in social tasks, and participants with a positive problem-solving orientation have a higher CPS ability than those with a negative problem-solving orientation*.

**H2.**  *Group sizes affect participants’ CPS ability in social tasks, and the CPS of individuals in tasks is significantly improved if they are provided with bigger groups*.

**H3.**  *There is a significant interaction effect between participants’ problem-solving orientation, size of the groups, and CPS ability; individuals show a higher level of CPS ability if they have a positive problem-solving orientation and are presented with large group sizes*.

### 4.2. Method

#### 4.2.1. Participants and Design

A total of 238 copies of Social Problem-Solving Inventory were randomly distributed online to students at a high school in Shandong Province in China; 224 valid questionnaires were collected. All participants were aged 15 years old (100 female; 123 male). Social Problem-Solving Inventory scores were standardized using the method proposed by Wang and Gu (2009) [15]. Thirty-six participants (22 female; 14 male) whose scores on positive problem-solving orientation and negative problem-solving orientation were higher than one standard deviation, were selected as the participants of experiment 1.

All participants had normal or corrected vision, had never participated in similar experiments before, and participated voluntarily. Participants were familiar with the use of Tencent QQ (PC version) and could type no less than 25 words per minute. Informed consent was obtained from participants and the study was approved by the Institutional Review Board of the university.

#### 4.2.2. Apparatus and Stimuli

**Measures of social problem-solving**: The problem-solving orientation of participants was measured using the Social Problem-Solving Inventory compiled by Wang and Gu (2009) [15]. The revised scale, from the original 52 questions that were reduced to 32 questions, adopted a five-point Likert scoring method, with “1” being representative of not being in complete conformity, and “5” being representative of character completely. Problem-solving orientation has two dimensions: positive tendency of four topics (i.e., When I have a problem, I believe it can be resolved); negative problem-solving orientation tends to be a total of five topics (i.e., when there is an important problem need to solve, I feel scared).

**The social tasks**: The social task in this study used a question compiled by PISA 2015. The question was: There are a group of foreign students who want to visit the area where the students take part in the test. The students who take part in the test need to make a visit plan with other students, negotiate the tour guide plan together, determine which students are the guides for which foreign students, and deal with emergencies during the visit. Because the testing system used in PISA 2015 is not open, this study adjusted the sample questions of PISA 2015, so the tasks and requirements of the questions remained unchanged while the tasks were adjusted in the context of Chinese culture.

**Instruments**: Tencent QQ and a timer. Tencent QQ is an internet-based instant messaging (IM) software developed by Tencent in 1999 based on ICQ, which allows communication in single and multiparticipant modes.

#### 4.2.3. Procedure

Bringing the screened participants into the laboratory individually, according to the pre-divided two-person group or three-person group, the participants were invited to sit and wait quietly, and the main test introduced the experimental process before the experiment formally started.

The experiment was conducted in a quiet and network-stable laboratory. After arriving at the laboratory, the participants were asked to carefully read the online experiment rules of CPS; then, the subjects were asked to use the Tencent QQ to complete the experiment, and they could not open any other program interface unrelated to the experiment during the experiment.

Participants completed a consent form with their basic information. The researcher created a discussion group in Tencent QQ, and the researcher and participants were added. There were three members, one researcher and two participants in the discussion group, who participated in the two-person groups; there were four members, one researcher and three participants in the discussion group, who participated in the three-person group. The researcher introduced the procedure, rules, and specific instructions of the experiment to participants, informing them that they would complete the experiment with the other (two or three) participants.

The researcher sent the task materials of the experiment to the discussion group. The participants in both two-person groups as well as three-person groups had 20 min to complete the CPS task. After the experiment, the researcher saved the data produced by each individual, removing it from the chat history before the next group started to keep the data sorted into relevant groups.

#### 4.2.4. Measurement

The social task of this study was formulated with reference to the sample questions of PISA 2015, and the evaluation of task results should also refer to the sample questions evaluation method of PISA 2015. PISA 2015 takes three core competencies of CPS and four problem-solving tasks at a personal level as the vertical and horizontal dimensions of the matrix, respectively, which constitutes 12 CPS-specific skills. According to the matrix of 12 CPS-specific skills, PISA divided the three dimensions of CPS ability (that is, to establish and maintain consensus; take appropriate actions to solve problems; establish and maintain group organization form) into three levels: low, medium, and high. Grading was performed using scores, where a low grade was one point, a medium grade was two points, and a high grade was three points. The three core competencies of CPS were obtained by adding the specific skill scores of the corresponding columns in the matrix.

The social tasks consisted of three specific tasks, including: (1) determine where foreign students are going to play, (2) determine who is the guide for foreign students, and (3) how to deal with emergencies. The three specific tasks were graded according to the grading standard of CPS core ability. 

Both raters were asked to rate a common set of 25% of the ideas to establish reliability. The reliability was satisfactory (the ICC value for the CPS was 0.877). Then, one rater was asked to rate the remaining ideas for novelty [28].

### 4.3. Results

#### 4.3.1. Descriptive Statistics

Experimental data were sorted and coded in Microsoft Excel. IBM SPSS Statistics 25.0 was used for data analysis including descriptive statistics, t-test, and ANOVA. In Experiment 1, the dependent variable index was the CPS ability of the participants. The results are shown in Table 1.

As can be seen from Table 1, regardless of whether the two-person group or the three-person group was considered, the CPS ability of individuals with a positive problem-solving orientation was clearly higher than that of individuals with a negative problem-solving orientation. For individuals with a positive problem-solving orientation, the performance of individuals in a three-person group was higher than that of individuals in a two-person group, while individuals with a negative problem-solving orientation were just the opposite, that is, the performance of individuals in a two-person group was higher than that of individuals in a three-person group.

#### 4.3.2. Interaction Analysis

A one-way ANOVA of participants’ gender with problem-solving orientation and CPS found that when the gender of participants was taken as an independent variable, there were some significant differences in various dimensions of problem-solving orientation (*t*(1, 34) = −2.319, *p* = 0.015 < 0.05). 

To control for the effect of gender on the results, a 2 (problem-solving orientation: positive and negative) × 2 (sizes of groups: small and large) between-group analysis of covariance (ANCOVA) was conducted, with problem-solving orientation and group sizes as independent variables, CPS ability as the dependent variable, and gender as a covariate. The results of ANCOVA are presented in Table 2.

Table 2 showed that after controlling for the gender of the participants, the main effect of problem-solving orientation on CPS ability was significant: *F* (1, 31) = 10.878, *p =* 0.002 < 0.05, *η*^2^*_p_* = 0.260. This suggests that problem-solving orientation can significantly affect the individual’s CPS ability. However, the main effect of group sizes on CPS ability was not significant: *F* (1, 31) = 0.021, *p* = 0.087 > 0.05. This suggests that group size does not affect the individual’s ability of CPS. The interaction effect between problem-solving orientation and group sizes was not significant on effectiveness: *F* (1, 31) = 0.890, *p* = 0.353 > 0.05. This suggests that problem-solving orientation had a direct effect on effectiveness and was moderated by group sizes. 

Further analysis showed that the score of the CPS core ability of individuals with a positive problem-solving orientation was much higher than that of individuals with a negative problem-solving orientation. For individuals with a positive orientation, the three-person group produced better CPS scores than those in the two-person group, but the results of this phenomenon were inconsistent for individuals with a negative problem-solving orientation. Therefore, in Experiment 1, the problem-solving orientation had a significant impact on the CPS ability scores of the participants, but there was no significant difference in group sizes.

### 4.4. Discussion

Experiment 1 investigated the CPS ability of participants under experimental conditions with different group sizes and different problem-solving orientations by controlling the size of groups presented in the experiment. We found that problem-solving orientation had a significant effect on CPS ability. The scores of individuals with a positive orientation were much higher than those with a negative orientation. However, in experiment 1, there was no significant effect of different group sizes on the ability of individual CPS.

Problem-solving orientation can promote the individual’s CPS ability, which accords with some expectations of Experiment 1 and supports existing research. The more positive the individual, the higher the score of their own CPS ability and the better the individual performance in the groups. This may be because individuals with a positive orientation believe more in their ability to solve problems in cooperation with others, so they are willing to put more effort into group work. However, individuals with a negative orientation do not think they have the ability to solve problems, so their mentality and cooperative attitude are negative, and they do not make a great effort in group cooperation. Wigfield and Eccles’ research in 2000 can confirm this view. They put forth the idea that the degree of individual effort depends on whether individuals think they have the ability to succeed [29]. The research of Dweck (2006) [30] also verifies that having a good growth mentality (affirmation of one’s own ability) has positive effects on efforts, persistence, and achievements. 

However, experiment 1 did not find a significant effect of group size on the ability of an individual’s CPS. However, it can provide a reference for future research and design. This result can mainly be explained from two aspects. First, this study divided the group sizes into two-person groups and three-person groups with reference to several studies, such as the measurement of CPS in PISA 2015 and the measurement of He (2019) [31]. These studies both measured the individual’s CPS ability in a systematic way, which is different from the online experimental form of this study. This shows that if online experiments are used to measure the individual’s CPS ability, it is necessary to consider the size of the groups. Second, in this study, the online experiment of CPS was conducted online, and there was no real contact between the participants. It is precisely because there was no real contact that the participants could only find the support of group partners from their “cold words,” which, to a certain extent, limits the performance of the participants [32]. 

## 5. Experiment 2

Experiment 1 found that the interaction between problem-solving tendency and group size was not significant. This suggests that only manipulating the problem-solving orientation cannot affect the CPS ability of individuals in different groups and that it is necessary to consider the effect of other individual factors. Therefore, experiment 2 will explore the effect of problem-solving style and group size on CPS ability by manipulating another dimension of problem solving (problem-solving style) and controlling the scale of individual participation in team size.

### 5.1. Hypotheses

Experiment 2 was intended to investigate the effect of problem-solving styles (rational and impulsive/neglect and evasion) and the number of group sizes (small and large) on CPS ability. If individuals can look at problems from a more rational perspective when facing tasks, they are more willing to take actions in tasks. However, if the individual’s cognition becomes unreasonable, the individual’s emotion will become negative and their behavior will deviate. We proposed the following hypotheses:

**H4.**  *Problem-solving styles affect participants’ CPS ability in social tasks, and participants with more rational problem-solving styles have a higher CPS ability than those with irrational (impulsive/neglect and evasion) problem-solving styles*.

**H5.**  *Group sizes affect participants’ CPS ability in social tasks, and the CPS of individuals is significantly improved if they are provided with bigger group in tasks*.

**H6.**  *There is a significant interaction effect between participants’ problem-solving styles, the number of group sizes, and CPS ability; individuals show a higher level of CPS ability if they have a rational problem-solving orientation and are presented with large group sizes*.

### 5.2. Method

#### 5.2.1. Participants and Design

A total of 238 copies of Social Problem-Solving Inventory were randomly distributed online to students at a high school in Shandong Province in China; 224 valid questionnaires were collected. All participants were aged 15 years old (100 female; 123 male). Social Problem-Solving Inventory scores were standardized using the method proposed by Wang and Gu (2009) [15]. Fifty-seven participants (29 female; 28 male) whose scores on the rational problem-solving styles and impulsive/neglect problem-solving styles and evasion problem-solving styles were higher than one standard deviation, were selected as participants for experiment 2.

All participants had normal or corrected vision, had never participated in similar experiments before, and participated voluntarily. Participants were familiar with the use of Tencent QQ (PC version) and could type no less than 25 words per minute. Informed consent was obtained from participants and the study was approved by the Institutional Review Board of the university.

#### 5.2.2. Apparatus and Stimuli

**Measures of social problem solving:** The problem-solving orientation of participants was measured using the Social Problem-Solving Inventory compiled by Wang and Gu (2009) [15]. The revised scale, from the original 52 questions that were reduced to 32 questions, adopted a five-point Likert scoring method, with “1” being representative of not being in complete conformity, and “5” being representative of character completely. The rational problem-solving style included 13 topics (i.e., when trying to solve problems, I often come up with a variety of methods and synthesize some of them to form better methods). There were six topics in the escaping from problem-solving style (i.e., when I encounter a problem in my life, I will delay solving it as much as possible) and the impulsive/negligent problem solving style consisted of four topics (i.e., when I need to make a decision, I will not consider the impact of each choice on others).

**The social tasks:** The same as in experiment 1.

**Instruments**: The same as in experiment 1.

#### 5.2.3. Procedure

Experiment 2 followed the same procedure as Experiment 1.

#### 5.2.4. Measurement

The measurement standard of CPS ability in experiment 2 was the same as that in experiment 1.

Both raters were asked to rate a common set of 25% of the ideas to establish reliability. The reliability was satisfactory (the ICC value for the CPS was 0.877). Then, one rater was asked to rate the remaining ideas for novelty [28].

### 5.3. Results

#### 5.3.1. Descriptive Statistics

Experimental data were sorted and coded in Microsoft Excel. IBM SPSS Statistics 25.0 was used for data analysis including descriptive statistics, t-test, and ANOVA. In Experiment 2, the dependent variable index was the CPS ability of the participants. The results are shown in Table 3.

As can be seen from Table 3, whether it was a two-person group or a three-person group, the CPS ability scores of individuals with a rational problem-solving style were significantly higher than those with an impulsive/negligent problem-solving style and an evasive problem-solving style. Compared with those who evade the problem-solving style, individuals with an impulsive/negligent problem-solving style had higher CPS ability scores. For individuals with a rational problem-solving style and an evasive problem-solving style, the performance of individuals in the group-scale cooperative of two-person groups was higher than that of individuals in three-person groups. However, the impulsive/negligent problem-solving style was just the opposite; the performance of the individual in the group-scale cooperative of the three-person group was higher than that of the two-person group. 

#### 5.3.2. Interaction Analysis

A one-way ANOVA of participants’ gender with problem-solving styles and CPS found that when the gender of participants was taken as an independent variable, there were some significant differences in various dimensions of CPS scores (*F* (1, 55) = 5.835, *p* = 0.019 < 0.05).

To control for the effect of gender on the results, a 3 (problem-solving styles: rational and impulsive/neglect and evasion) × 2 (sizes of groups: small and large) between-groups analysis of covariance (ANCOVA) was conducted, with problem-solving styles and group sizes as independent variables, CPS ability as the dependent variable, and gender as a covariate. The results of ANCOVA are presented in Table 4.

Table 4 shows that after controlling for the gender of the participants, the main effect of problem-solving styles on CPS ability was significant: *F* (1, 50) = 32.214, *p <* 0.001, *η*^2^*_p_* = 0.563. It suggests that problem-solving styles can significantly affect the individual’s CPS ability. 

Based on Table 4, the main effect of group sizes on CPS ability was significant: *F* (1, 50) = 4.634, *p* = 0.036 < 0.05, *η*^2^*_p_* = 0.085. This suggests that group size can also significantly affect the individual’s ability of CPS. 

The interaction effect between problem-solving orientation and group sizes was not significant on effectiveness: *F* (1, 50) = 0.890, *p* = 0.094 > 0.05. This suggests that problem-solving style was not moderated by group size, and group size was not moderated by problem-solving style.

In sum, in Experiment 2, both problem-solving style and group size had an impact on the individual’s CPS ability, but one was not regulated by the other. In other words, problem-solving style was not regulated by group size, and group size was not regulated by problem-solving style.

Further analysis showed that the scores of rational-style individuals in CPS ability were much higher than those with an impulsive/negligent style and an evasive style. For individuals with a rational style and an evasive style, the three-person groups could produce better CPS scores than the two-person groups could, but this phenomenon was inconsistent in impulsive/negligent-style individuals. Therefore, in experiment 2, the problem-solving styles had a significant impact on the CPS ability scores of the subjects, and the group size could also significantly affect the CPS ability scores of individuals, but there was no moderating effect between them.

### 5.4. Discussion

Experiment 2 investigated the CPS ability of participants under experimental conditions with different group sizes and different problem-solving styles by controlling the size of groups presented in the experiment. We found that problem-solving styles had a significant effect on CPS ability. The scores of individuals with more rational problem-solving styles were much higher than those with irrational problem-solving styles. However, in experiment 2, there was no significant interactive effect of problem-solving styles and group sizes on CPS ability. 

Based on experiment 2, we found that problem-solving style had a significant effect on the individual’s CPS ability, which is in line with some expectations of experiment 2 and is also a confirmation supplement to previous research results. Compared with the escapist problem-solving teenagers and impulsive/negligent problem-solving teenagers, the CPS ability of rational problem-solving teenagers was higher. Cognitive behavioral theory can help explain the results of this study. A series of emotional and behavioral problems caused by people are not caused by events themselves, but by people’s interpretation and evaluation of events. That is to say, excluding things themselves, if individuals can look at problems from a more rational perspective when facing tasks, they are more willing to take actions in tasks and try their best to solve problems in tasks. However, if the individual’s cognition becomes unreasonable (for example, the cognition of evasion and negligence appears), the individual’s emotion will become negative and their behavior will deviate, and they will be unwilling to cooperate with other members to solve problems in cooperative tasks.

Consistent with previous research results, experiment 2 found that group size significantly affected the performance of an individual’s CPS ability. As Dennis and Valacich reported in their research in 1993, large groups have more positive effects on individuals than small groups [33]. The advantage of a group is that the ideas shared by different members can be used as the knowledge reserve put forward by individuals for their ideas [34]. In addition, the ideas of group members can stimulate individuals to produce ideas that were not previously available in related fields [35]. This means that in large groups, individuals are more likely to have access to other people’s ideas, strengthen their ability to cooperate and solve problems, and enjoy the process of cooperation more fully, thus promoting their personal performance in the group. Furthermore, the research of Paulus and Yang (2000) [36] also showed that if individuals are motivated to pay attention to shared ideas or viewpoints, the effect of these existing ideas and answers on individuals will be enhanced. This study emphasizes the importance of cooperation and requires the participants and group members to complete the CPS task together. In this process, individuals should constantly communicate with others and think about other people’s ideas and viewpoints; it is this process that strengthens the positive effect of shared ideas on individual performance. 

## 6. General Discussion

The present study explored the effect of problem-solvers’ characteristics and group sizes and their interaction effect on the CPS ability of individuals through two online experiments. The experiments were intended to answer two major questions: (1) How do problem-solvers’ characteristics affect participants’ CPS ability in a social task? (2) What is the role of group sizes in such a relationship?

In our results, problem-solvers’ characteristics (problem-solving orientation and problem-solving styles) were found to be conducive to the CPS ability of individuals. In addition, group size was found to have some effect on the CPS ability of individuals. However, an interaction effect between intrinsic motivation and the quantity and novelty of cues on CPS ability was not found.

This paper verified that better problem-solvers’ characteristics can promote individuals to perform better in CPS. In experiment 1, it was found that problem-solving orientation significantly promoted the CPS core ability of individuals, and the score of the CPS core ability of individuals with a positive problem-solving orientation was much higher than that of individuals with a negative problem-solving orientation. The explanation of “positive problem-solving orientation” and “negative problem-solving orientation” by D’Zurilla and Nezu (2010) [37] can help us confirm this. Individuals with a positive problem-solving orientation believe that they can solve problems even if they feel those problems are difficult. They believe that group members can solve tasks together, and they have a stronger sense of self-efficacy and positive behavior cognition. When they encounter problems, their emotions are optimistic and clear, instead of being passive and choosing to avoid problems. On the contrary, individuals with a negative orientation are more “complaining” in their attitude when they encounter things. Most of the time, they think they cannot solve problems. Even if the group cooperates, everyone cannot solve problems through cooperation. Individuals with a negative problem-solving orientation are more likely to have negative emotions and cognitive behavioral deviations.

In experiment 2, the problem-solving style significantly promoted the ability of an individual’s CPS. Individuals with a rational problem-solving style scored much higher in the ability of CPS than those with an impulsive/negligent problem-solving style or an evasive problem-solving style. D’Zurilla and Nezu (2010) [37] pointed out in their theoretical interpretation of “rational problem-solving style” that individuals with a rational style are more willing to adopt a comprehensive and systematic way to solve problems, which can encourage individuals to think deliberately, only immerse themselves in problems and discussions with group members, and aspire to obtain correct solutions through effort. The research of McGuire (2005) [38] also confirmed this view. When D’Zurilla and Nezu (2010) [37] mentioned the “impulsive/negligent problem-solving style,” they explained that most of these individuals would not seriously consider problems when they encountered them, and usually only chose the first scheme and did not consider whether the scheme was really feasible, which was prone to “perfunctory things.” Those individuals who like to avoid problems are the opposite of rational individuals; when they encounter problems, their first reaction is to escape. They do not want to solve the problem, and they even turn a blind eye and pretend that they do not have a task [37].

Although the effect of group size on individual CPS ability was not found in experiment 1, the main effect of group size on individual CPS ability was significant in experiment 2. When an individual is in a multi-crew group, their CPS ability is better. This is because the number of ideas members have increase in larger group sizes, and the ideas of other members can stimulate the remote nodes in the original semantic network of individuals [39]. According to the activation diffusion model, when an individual node is activated, other closely connected nodes will be activated accordingly [40]. In short, the more ideas the group members have, the more active the individual nodes will be, and the better their performance will be. 

In summary, this study conducted two online experiments on CPS. The results show that better problem-solvers’ characteristics can result in individuals’ better CPS performance, and group size can also affect individuals’ CPS ability. 

The above conclusions are of great practical significance to the improvement of middle school students’ CPS ability, especially the change in CPS attitude and thinking mode. In the future, educators can set different forms of cooperation for individuals with different problem-solvers’ characteristics and improve their CPS ability through training and practice.

## 7. Limitations and Future Directions

This research has a few limitations. First, the CPS task was conducted online, and the performance of CPS ability may be reduced due to “evaluation concerns.” In addition, the participants could only complete the experimental tasks through online cooperation instead of communication in real situations, which may lead to the phenomenon of “words fail to reach the meaning.” Compared with laboratory experiments, this experiment is closer to the real CPS task and has higher ecological validity, but future research needs to further balance the effect of ecological validity and interference factors on the experiment by perfecting experimental rules.

Second, this study divided the group sizes into two-person groups and three-person groups. Although it refers to the paradigm provided in previous research, no significant effect of group size on cooperative problem-solving ability was found in the first study, indicating that there may be other criteria for the division of group sizes. Group size has been a regular focus of research. In the research of the creative field, most researchers divide groups into three-person groups and nine-person groups. Previous studies found that the larger the number of participants in the group, the more creative ideas the participants produced [35,41,42]. Similarly, the number of members can affect the performance of an individual’s CPS, and group size and group type can be the focus of future research.

Third, this study refers to the age choice of participants in PISA, so only 15-year-old teenagers were selected as the research object. Due to the limitation of sampling, this study only conducted experiments from senior students in a certain area, so it may not be extended to other grades or sections. Future research can explore the effect of social problem solving and team size on adolescents’ cooperative problem-solving ability from multiple sections.

Finally, this study comprehensively provided an understanding of the effect of problem-solvers’ characteristics and group size on CPS ability. However, because of the relative independence of each index, we ultimately received the overall evaluation of CPS ability rather than a complete understanding of the cooperative process and the problem-solving process. Future research can consider a variety of task types or set different task difficulties in similar tasks. “Cooperation” and “problem solving” are two dimensions of CPS, and the thinking process of participants in these two processes and the generation of “cooperation” and “problem solving” can also be the focus of future research.

## 8. Conclusions

This study found that different groups have a different effect on CPS ability. Although this study only found the role of group size in the effect of problem-solving style and group size on teenagers’ CPS ability, it is probable that the larger the group size is for an individual, the better their performance of CPS will be. This study also enriches the research results of group size in the field of CPS. It provides a research direction on how to promote the improvement of an individual’s CPS ability, and underlines the necessity to cultivate an individual’s problem-solving characteristics. Educators can set different types of cooperation forms for individuals with different characteristics of problem solvers, and improve their CPS ability through training and practice. 

## Figures and Tables

**Table 1:** CPS scores under different problem-solving orientations and group sizes (M ± SD).

Group Sizes	Positive Problem-Solving Orientation	Negative Problem-Solving Orientation
	CPS (M ± SD)	CPS (M ± SD)
two-person groups	18.091 ± 1.514	14.429 ± 2.992
three-person groups	19.222 ± 6.924	12.889 ± 3.100
Total score	18.600 ± 4.661	13.563 ± 3.054

Note. M = mean, SD = standard deviation.

**Table 2:** Variance analysis of CPS scores under different problem-solving orientations and team sizes.

Source of Variance	*df*	*MS*	*F*	*p*	*η* ^2^
problem-solving orientation (a)	1	188.457	10.878 *	0.002	0.260
Group sizes (b)	1	0.357	0.021	0.887	0.001
a × b	1	15.418	0.890	0.353	0.028
Deviation	31	17.324			

Note. * *p* < 0.05.

**Table 3:** CPS scores under different problem-solving styles and group sizes (M ± SD).

Group Sizes	Rational Problem-Solving Style	Impulsive/Negligent Problem-Solving Style	Evasive Problem-Solving Style
	CPS (M ± SD)	CPS (M ± SD)	CPS (M ± SD)
two-person groups	20.800 ± 3.676	13.875 ± 3.907	11.100 ± 2.846
three-person groups	17.200 ± 3.521	11.500 ± 2.593	11.778 ± 1.302
Total score	19.000 ± 3.960	12.556 ± 3.365	11.421 ± 2.219

Note. M = mean, SD = standard deviation.

**Table 4:** Variance analysis of CPS scores under different problem-solving styles and team sizes.

Source of Variance	*df*	*MS*	*F*	*p*	*η* ^2^
problem-solving style (a)	2	312.329	32.214 ***	0.000	0.563
Group sizes (b)	1	44.928	4.634 *	0.036	0.085
a × b	2	24.030	2.478	0.094	0.090
Deviation	50	9.695			

Note. * *p* < 0.05; *** *p* < 0.001.

## Data Availability

The data presented in this study are available on request from the corresponding author.
